# Hemoglobin levels and anemia evaluation during pregnancy in the highlands of Tibet: a hospital-based study

**DOI:** 10.1186/1471-2458-9-336

**Published:** 2009-09-15

**Authors:** Yuan Xing, Hong Yan, Shaonong Dang, Bianba Zhuoma, Xiaoyan Zhou, Duolao Wang

**Affiliations:** 1 Department of Epidemiology and Health Statistics, Xi'an Jiaotong University College of Medicine, Xi'an, PR China; 2 Obstetrics and Gynecology Department, Lhasa People's Hospital, Lhasa, PR China; 3 Department of Epidemiology and Population Health, London School of Hygiene and Tropical Medicine, London, WC1E 7HT, UK

## Abstract

**Background:**

Anemia is regarded as a major risk factor for unfavorable pregnancy outcomes, but there have been no previous studies describing the pattern of hemoglobin concentration during pregnancy in Tibet and the relationship between altitude and Hb concentration in the pregnant women living in Tibet still has not been clearly established. The main objectives of this study were to study the hemoglobin levels and prevalence of anemia among pregnant women living in the highlands of Tibet and to evaluate potential associations of hemoglobin and anemia with women's characteristics.

**Methods:**

The hospital-based study was conducted in 380 pregnant women. Their blood samples were tested and related sociodemographic information was collected. Multiple linear regression model and multiple logistic regression model were used to assess the association of pregnant women's characteristics with hemoglobin level and the occurrence of anemia. Centers for Disease Control (CDC), Dirren et al. and Dallman et al. methods were used to adjust the hemoglobin measurements based on altitude for estimating the prevalence of anemia.

**Results:**

The mean hemoglobin concentration was 127.6 g/L (range: 55.0-190.0 g/L). Prevalence rate of anemia in this study was 70.0%, 77.9% and 41.3%, respectively for three altitude-correction methods for hemoglobin (CDC method, Dirren et al. method, and Dallman et al. method). Gestational age, ethnicity, residence and income were significantly associated with the hemoglobin concentration and prevalence of anemia in the study population. Specially, the hemoglobin concentration of pregnant women decreased with increase in gestational age.

**Conclusion:**

The hemoglobin level was low and prevalence rate of anemia was high among pregnant women in Lhasa, Tibet. Gestational age, ethnicity, residence and income were found to be significantly associated with the hemoglobin level and the occurrence of anemia in the study population.

## Background

Anemia is a critical health concern because it affects adversely growth and energy levels. Anemia afflicts about two billion people worldwide, largely due to iron deficiency [1]. It damages immune mechanisms and is also associated with increased morbidity. Anemia is regarded as a major risk factor for unfavorable pregnancy outcomes. It has been associated with premature labor [2], low birth weight [3,4], maternal mortality [5], and perinatal mortality [6]. It is estimated that more than half of pregnant women in developing countries suffer from anemia [1]. The definition of anemia can be based on either hemoglobin or hematocrit concentration [7]. According to the World Health Organization (WHO), anemia in pregnancy is defined as hemoglobin (Hb) concentration of less than 110 g/L [1].

Hb has the physiological function of carrying oxygen, and accordingly, environmental oxygen tension can affect Hb concentration in the blood. Environmental oxygen declines gradually with increasing altitude. Research has shown that Hb increases with an increase of altitude especially when the altitude is above 1000 m above sea level [8-10]. Understanding the relationship between Hb concentration of pregnant women living in Tibet and altitude will have important implications for correctly estimating the prevalence of anemia at high altitudes.

The Qinghai-Tibet Plateau, with an average altitude of 4500 m, has an area of about 2.5 million km^2^, accounting for a quarter of the land in China. It is the highest plateau on earth, and is often referred to as "the roof of the world". The relationship between altitude and Hb concentration during pregnancy in this high altitude area still has not been clearly established, and there have been limited studies describing Hb concentration during pregnancy. Our primary objectives were to study the pattern of hemoglobin level and prevalence of anemia among pregnant women living in the highlands of Tibet and to evaluate effects of women's demographic and socioeconomic characteristics on hemoglobin level and the occurrence of anemia. Our secondary objectives were to assess the sensitivity of the estimates of anemia to three altitude-correction methods for hemoglobin (CDC method, Dirren et al. method, and Dallman et al. method) and to examine the associations between hemoglobin and erythrocyte parameters.

## Methods

### Setting

The study was conducted in Lhasa, the capital of Tibet, which is located on the Qinghai-Tibet Plateau. The mean altitude of Lhasa is 3685 m above sea level. Eighty-six percent of this plateau has harsh natural environments such as decreased oxygen partial pressure and cold temperatures. The population census showed that in 2003 there were about 2.59 million inhabitants in Tibet, and 96% of them belonged to the Tibetan nationality [11]. The rural population accounted for about 85% of the total population. Farming and animal husbandry are the main economic activities [12]. The Lhasa People's Hospital is one of main hospitals which provide antenatal care for women living in Lhasa city and the number of women attending antenatal care at the Obstetrical Department of the Lhasa People's Hospital is about 1200 per year.

### Participants

This study was a cross-sectional, hospital-based study with randomly selected pregnant women who lived in Lhasa. During one-year investigating period, we randomly selected 2 days a week considering seasonal variation, on which all the pregnant women who sought antenatal care in the obstetrical department of Lhasa People's hospital were selected as study subjects. They were interviewed face-to-face by trained professional interviewers and their Hb levels were measured at the same time. In addition, we randomly selected a subgroup from all participants to undergo laboratory tests for their erythrocyte parameters. Selection procedures of the subgroup were done as following: first, all the participants on the selected day were sequentially numbered from 1 to the number for the last participant on that day and secondly all the participants with either an odd or even number were chosen depending whether a random number was 1 or 2 (one for odd number and 2 for even number). A questionnaire was used to collect each participant's name, address, age, ethnicity, income, and gestational age. Exact gestational age was difficult to determine for some women because they did not know their dates of last menstrual period and if so the month of their last menstruation was recorded as an approximate gestational age.

The study was approved by the Ethic Review Committee and Academic Committee, Xi'an Jiaotong University College of Medicine, Xi'an, China. Pregnant women who agreed to participate in the study signed the consent form.

### Sample size

As an exploratory investigation, this study recruited 396 pregnant women between October 2006 and September 2007. Of them, 2 were later found to be not pregnant, and 14 did not agree to have their Hb levels checked due to fear of pain, religious beliefs. In total, the Hb concentrations were available for 380 (96.0%) pregnant women, which formed the sample of this study. Due to limited resources, we planned to test erythrocyte parameters for half of the sample. However, some pregnant women didn't want to have their finger prick twice. As a result, we only obtained blood samples for 106 women, who were further tested for red blood cells (RBC), mean corpuscular volume (MCV), red cell distribution width (RDW) and other erythrocyte parameters.

### Measurement of Hb and erythrocyte parameters

Capillary blood was collected from each participant using a finger-prick method to extract three drops of blood from the left middle and/or ring finger. The first and second drops of blood were wiped away. The third drop was used immediately for testing Hb. The third drop of blood from the other finger was used for testing other erythrocyte parameters. Hb concentration was measured by Hb photometer (B-Hemoglobin, precision of 1 g/L, HemoCue AB, Sweden). Erythrocyte parameters were measured by hematology analyzer (Sysmex KX-21N, Japan). The definition of anemia used in this analysis was an Hb concentration value of less than 110 g/L [1]. The reference of erythrocyte parameters were [13]: RBC (10^12^/L): 3.5-5.5; HCT (l/L): 0.37-0.48; MCV (fL): 82-95; MCH (pg): 27-31; MCHC (g/L): 320-360; RDW-CV: 0.11-0.16.

### Hb adjusted for altitude

Three altitude correction methods, the CDC [8], Dirren et al. [9] and Dallman et al. [10], were used in our study to adjust the hemoglobin concentration for altitude. They can be expressed as: CDC: ΔHb = -0.032×(Alt)+0.022×(Alt)^2^, where δHb is the increment of Hb by increased altitude above sea level, and Alt is the altitude (1000 m×3.3); Dirren et al.: Hb_sea-level _= Hb_measured_-3.44×(e ^(0. 000 633×Alt)^-1), where Hb_sea level _stands the concentration after adjustment, and Alt for altitude (m); and in Dallman et al.: Hb increased by 4 g/L per 1000 m elevation above sea level.

### Data analysis

Multiple linear regression model and multiple logistic regression model were used to assess the influences of women's characteristics on hemoglobin level and the occurrence of anemia. In both regression analysis, we defined ethnicity, residence and income as follows: Ethnicity = 0 for a Tibetan and 1 for a non-Tibetan; Residence = 0 for a woman living in urban area and 1 in rural area; Annual income = 0 if annual income<$264 and 1 otherwise. Gestational age, maternal age and parity were analyzed as continuous variables in the model. Gestational age was log transformed before analyses as the transformed gestational age fits the data better.

Log transformation was done on these parameters to normalize their distributions. The proportion classified as anemia after adjustment was used for assessing agreement of three altitude correction methods. One-sample t-test was used to compare mean of an erythrocyte parameter with its reference and the difference between the mean and reference together with its 95% CI for a parameter was also derived.

SPSS Version 8.0 (Statistical Package for Social Science, Inc., Chicago, USA) was used for statistical analysis. All statistical tests were two-tailed with a significance level of 0.05.

## Results

### Characteristics and hemoglobin concentration of sample

**Figure 1:**
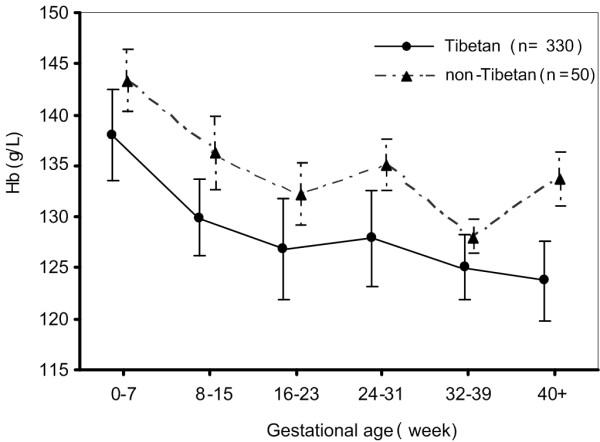
**Mean hemoglobin concentration (± standard error) at different gestational age groups among 380 pregnant women in Lhasa, Tibet**.

Of the 380 pregnant women included in this study, 86.8% were Tibetan. The mean age of the women was 26.9 years, ranging from 16 to 36 years. Rural dwellers accounted for 38.4% of the participants (Table 1), and background characteristics were found to be similar between Tibetan and non-Tibetan (p > 0.05 for each variable). In all, the mean Hb concentration was 127.6 g/L (s.d.:19.8 g/L) with a range from 55.0 to 190.0 g/L: 126.6 g/L (s.d.:20.2 g/L) for Tibetans, 134.6 g/L (s.d.:15.1 g/L) for non-Tibetans, 130.4 g/L (s.d.:20.9 g/L) for rural women and 125.9 g/L (s.d.:18.9 g/L) for urban women, 119.8 g/L (s.d.:22.6 g/L) for annual income less than $264 and 128.8 g/L (s.d.:19.5 g/L) for annual income more than $264, respectively. Relationship between Hb and gestational age by ethnicity is shown in Figure 1. The figure shows that the Hb decreased non-linearly with the increase in gestational age for both Tibetan and non-Tibetan women and that the Hb for Tibetan women was consistently lower than for non-Tibetan.

**Table 1:** Characteristics, hemoglobin concentration and correlates of Hb of study sample (g/L)

			**Multiple linear regression analysis^d^**	
**Characteristics**	**n (%)**	**Mean ± s.d. (95% CI) of Hb (g/L)**	**Coeff (95% CI)**	***P*-value**
Gestational age^a^			-0.25 (-0.44, -0.05)	0.012
1st trimester	49 (12.9)	135.8 ± 20.9 (132.2, 143.8)		
2nd trimester	119 (31.3)	128.0 ± 21.0 (124.4, 132.2)		
3rd trimester	212 (55.8)	125.6 ± 18.3 (123.3, 128.5)		
Age			-0.14 (-0.59, 0.30)	0.524
<25	123 (32.4)	128.6 ± 20.0 (125.4, 132.9)		
25-30	163 (42.9)	128.0 ± 19.1 (125.4, 131.5)		
30+	93 (24.5)	125.7 ± 20.7 (122.3, 130.9)		
Ethnicity			8.09 (1.68, 14.50)	0.013
Tibetan	330 (86.8)	126.6 ± 20.2 (124.9, 129.4)		
Non-Tibetan^b^	50 (13.2)	134.6 ± 15.1 (130.8, 139.5)		
Residence			5.66 (1.03, 10.30)	0.017
Urban	234 (61.6)	125.9 ± 18.9 (124.1, 129.1)		
Rural^c^	146 (38.4)	130.4 ± 20.9 (127.3, 134.4)		
Parity			1.23 (-1.72, 4.17)	0.414
0	173 (45.5)	127.4 ± 19.1 (124.7, 130.6)		
1	120 (31.6)	126.4 ± 21.2 (123.0, 130.9)		
2+	87 (22.9)	130.5 ± 18.9 (126.8, 135.2)		
Annual income ($)			10.65 (3.04, 18.25)	0.006
<264	49 (12.9)	119.8 ± 22.6 (111.0, 128.5)		
264+	331(87.1)	128.8 ± 19.5 (126.8, 131.0)		

Total	380 (100.0)	127.6 ± 19.8 (125.7, 129.6)		

^a^Gestational age is in logarithmic scale

^b^Non-Tibetan include Han, Hui, Menba, Luoba and Naxi nationalities residing permanently in Tibet

^c^Rural includes agricultural and pastoral areas

^d^Coeff: regression coefficient; CI: Confidence Interval

### Correlates of hemoglobin

The results from multiple linear regression analysis of Hb are presented in Table 1. The table shows that Hb was associated with log gestational age (Coeff = -0.25, 95% CI: -0.44, -0.05). Ethnic Tibetans had lower Hb level than non-Tibetans (Coeff = 8.09, 95% CI: 1.68, 14.50). Pregnant women living in rural area had higher hemoglobin level than those in urban area (Coeff = 5.66, 95% CI: 1.03, 10.30). Pregnant women with annual income less $264 had significantly lower Hb than those with annual income ≥ $264 (Coeff = 10.65, 95% CI: 3.04, 18.25).

### Erythrocyte parameters

We observed that the study sample of 380 women and its subgroup of 106 women with blood samples are similar in terms of the observed characteristics in Table 1 (p value> 0.05 for each variable). When means of erythrocyte parameters' 95%CI were calculated, we found that 95% CI of all parameters (RBC: mean: 3.89, 95%CI: 3.77-4.01 10^12^/L; HCT: mean: 0.37, 95%CI: 0.35-0.39 l/L; MCV: mean: 87.37, 95%CI: 80.8-93.8 fL; MCHC: mean: 334.70, 95%CI: 313.7-365.2 g/L; RDW-CV: mean: 0.15, 95%CI: 0.14-0.19) were contains the upper limit or lower limit of references except for MCH (32-43 pg). Mean of MCH was higher than that of the sea level pregnancy groups (mean: 35 pg, P < 0.01).

### Consistency of the three methods for evaluating anemia

**Figure 2:**
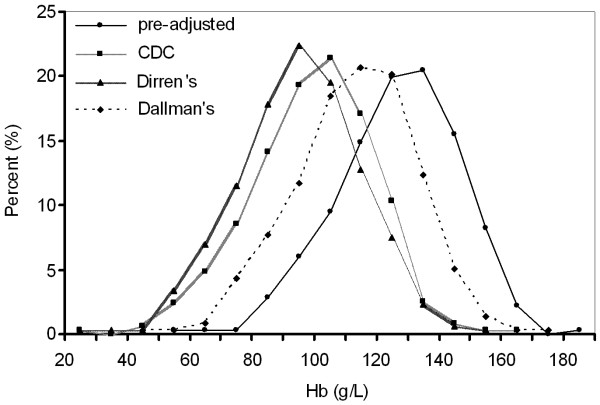
**Hemoglobin distribution among 380 pregnant women attending antenatal clinics in Lhasa, Tibet, according to three altitude adjustment methods**.

Before adjustment for altitude, the percentage of women with Hb being less than 110 g/L was 16.6%, 18.5% for Tibetan women and 4.0% for non-Tibetan women. The three adjusted distribution curves are plotted in Figure 2, with the Dirren et al. curve shifting to the far left. The Dallman et al. curve was the closest to the pre-adjusted curve. The corresponding prevalence rate of anemia adjusted for altitude was 70.0%, 77.9% and 41.3% for the CDC method, the Dirren et al. method and the Dallman et al. method, respectively. The above results suggest a good consistency for evaluating anemia adjusting for altitude between CDC and Dirren et al. methods but poor consistency between CDC and Dallman et al. methods, and very poor consistency between Dirren et al. and Dallman et al. methods.

### Correlates of anemia

To assess the correlates of anemia, we used the CDC correction method for determining an anemia. The multiple logistic regression results are presented in Table 2. The table shows that Tibetan pregnant women were more likely to have an anemia than non-Tibetan pregnant women (OR= 0.45, 95% CI: 0.23, 0.88). Pregnant women living in urban area or those of annual income less than $264 were also more likely to have an anemia (OR for residence = 0.39, 95% CI: 0.24, 0.69; OR for annual income = 0.37, 95% CI: 0.16, 0.84).

**Table 2:** Distribution of anemia (after CDC altitude adjustment) by characteristics of women and odds ratios of anemia from multiple logistic regression model analysis (N = 380)

		**Multiple logistic regression analysis**^d^	
**Characteristics**	**Prevalence of anemia (%) (95%CI)**	**odds ratio (95% CI)**	***P*-value**
Gestational age^a^		1.89 (1.25, 2.85)	0.003
1st trimester	53.1 (38.6, 67.5)		
2nd trimester	66.4 (57.8, 75.0)		
3rd trimester	75.9 (70.1, 81.7)		
Age		1.30 (0.91, 1.85)	0.166
<25	66.7 (58.2, 75.1)		
25-30	70.3 (63.3, 77.5)		
30+	73.1 (63.6, 82.1)		
Ethnicity		0.45 (0.23, 0.88)	0.006
Tibetan	71.2 (66.2, 76.0)		
Non-Tibetan^b^	60.4 (46.1, 74.8)		
Residence		0.39 (0.24, 0.69)	0.004
Urban	75.2 (69.3, 80.5)		
Rural^c^	61.6 (53.7, 69.6)		
Parity		0.79 (0.57, 1.11)	0.170
0	71.3 (64.5, 78.2)		
1	72.5 (64.4, 80.6)		
2+	63.2 (52.4, 73.2)		
Annual income ($)		0.37 (0.16, 0.84)	0.011
<264	82.1 (71.8, 94.1)		
264+	68.0 (62.8, 72.9)		

Total	70.0 (65.4, 74.6)		

^a^Gestational age is in logarithmic scale

^b^Non-Tibetan include Han, Hui, Menba, Luoba and Naxi nationalities residing permanently in Tibet

^c^Rural includes agricultural and pastoral areas

^d^Outcome in the logistic regression model defined as 0 for Hb ≥ 110 and 1 for Hb<110

## Discussion

This hospital-based study conducted in Lhasa showed low Hb concentration among pregnant women. The average hemoglobin concentration was 127.6 g/L, lower than the 137.1 g/L among those residing at about the same altitude (3600 m) in Bolivia [14]. The Hb level for most of the participants in our study was lower than the WHO criteria values related to long-term high-altitude exposure [1].

Our study showed that pregnant Tibetans had lower Hb levels than non-Tibetans. The Tibetans are the aboriginal inhabitants in Tibet with a long history [15]. Some physical adaptations have induced specific changes in Hb with increasing altitude. For example, Tibetans had higher vital capacity, thus can increase capacity to move more oxygen through the lungs which might be expected to result in more oxygen in the bloodstream [16,17]. Another important physiological feature found particularly among Tibetans was a denser capillary network [18]. It could make more thorough exchange of oxygen. As per the above adaptations, we suggested that Tibetans have an improved capability for accommodating the low-oxygen circumstances without having an increased concentration of hemoglobin. However, most of the non-Tibetans in the region were immigrants whom from low-lying provinces and their physiological adaptation for environment of high altitude might be different from the Tibetan, which could contribute to the ethnic differences in Hb level and prevalence of anemia to some extent. This needs further investigation.

A previous study has shown a U-shaped relationship between Hb and gestational age [19], which is generally attributed to physiological hemodilution. Hb levels appear to be lowest when dilution is at its maximum (at seven to eight months' gestation). This study showed that Hb level among pregnant women living in Tibet decreased with increase in gestational age. The Hb of urban pregnant women was lower than that of rural pregnant women. Similar finding was found in a large study in China in 1992 [20,21], but the reasons for such a finding are unclear.

According to Bessman et al.'s study [22], iron deficiency anemia cases show specific erythrocyte changes, such as an MCV decrease and RDW-CV increase. In our study, although the prevalence of anemia was 70.0% (CDC method's), but neither MCV nor RDW-CV showed any change. As such, we could not conclude that the population in our study suffered iron deficiency anemia. MCH indicates the average hemoglobin concentration in each erythrocyte. The increase of MCH may suggest a physiological adaptation differences in this population.

Using three different altitude correction procedures (CDC method, Dirren et al. method, and Dallman et al. method), we estimated the prevalence rate of anemia being 70.0%, 77.9% or 41.3%, respectively, in contrast with 19.5%, 13.6% and 28.9% for pregnant women in Beijing [23], Shanghai [24] and other part of China [21]. Although the anemia was quite high among the pregnant women in our study, most of anemic women were not with typical clinical symptoms of anemia. For example, in all 380 women included in this study, only 14% had frequent dizziness, 18% reported lack of physical strength, and about 8% were found to have pallid palpebral conjunctiva. Therfore, we concluded that these three methods were probably not suitable for correcting Hb for altitude for pregnant women living in the highlands of Tibet. The most important reason for this may be that the altitude ranges for those three methods were all between 0 to 3200 meters, while the participants in this study were living in the altitude of 3680 m. So extrapolations of these three methods may lead to unreliable results. The second reason may be that different methods used different fitting curves. The CDC's is conic, while that for the above-3000 m results is steep. Dirren et al. is an exponential curve. Although it has a gentle slope, it had a small sample size at each altitude. In comparison, Dallman et al. is a straight line. Other previous studies have shown that Hb did not increase linearly with higher altitudes [14,25]. However, so far it is still unclear what relationship between Hb and altitude for Tibetan women is.

Using a survey dataset on nutritional status of younger children living on the Qinghai-Tibet Plateau, Dang et al showed a positive association between Hb concentrations and altitude, but that the results did not contrast dramatically with these from the CDC, Dirren et al. and Dallman et al. methods [25]. The clear relationship between altitude and Hb concentration has still not been established yet in pregnant women living in the Qinghai-Tibet Plateau. As such, we applied three available methods to assess the prevalence of anemia but found very inconsistent prevalence rates. By comparing the consistency among these three methods, we concluded that the CDC method might be slightly better until a new hemoglobin-altitude formula for people living at this altitude can be established.

This is the first study that described the pattern of Hb concentration and prevalence of anemia among pregnant women living in the highlands of Tibet. The hospital-based cross-sectional design and selection bias of the sample in this study may underestimate the real prevalence of anemia and may not provide direct epidemiological inference for causality. However, our study serves as useful information and indicates a need for further anemia studies and interventions for pregnant women in the highlands of Tibet. A smaller sample size among the first-trimester pregnancies, due to some participants' restrictive traditional beliefs, may have introduced bias into this study. Lack of measurements of serum indexes such as serum ferritin and transferrin receptor may have limited our understanding of the true prevalence of anemia. In addition, the results from this hospital-based study may be subject to unobserved confounding factors, which need further investigation.

## Conclusion

The hemoglobin level was low and prevalence rate of anemia was high among pregnant women in Lhasa, Tibet. Gestational age, ethnicity, residence and income were found to be significantly associated with the hemoglobin level and the occurrence of anemia in the study population. The three available altitude-correction methods for hemoglobin might have over-estimated the prevalence of anemia for pregnant women living in Tibet. The relationship between altitude and Hb for pregnant women living in the Tibetan plateau requires further study in order to determine accurately the magnitude of anemia during pregnancy.

## Competing interests

The authors declare that they have no competing interests.

## Authors' contributions

HY conceptualized the study. YX was involved in the design and implementation of the study. SD and YX were responsible for field work and completed the analyses. YX synthesized analyses and led the writing. BZ and XZ assisted with the field work. DW provided important suggestions on study design and data analyses and reviewed drafts of the article. All authors read and approved the final manuscript.

## Pre-publication history

The pre-publication history for this paper can be accessed here:


